# The *Actinobacillus pleuropneumoniae* HMW1C-Like Glycosyltransferase Mediates N-Linked Glycosylation of the *Haemophilus influenzae* HMW1 Adhesin

**DOI:** 10.1371/journal.pone.0015888

**Published:** 2010-12-30

**Authors:** Kyoung-Jae Choi, Susan Grass, Seonghee Paek, Joseph W. St. Geme, Hye-Jeong Yeo

**Affiliations:** 1 Department of Biology and Biochemistry, University of Houston, Houston, Texas, United States of America; 2 Department of Pediatrics and Department of Molecular Genetics and Microbiology, Duke University Medical Center, Durham, North Carolina, United States of America; University of Delhi, India

## Abstract

The *Haemophilus influenzae* HMW1 adhesin is an important virulence exoprotein that is secreted via the two-partner secretion pathway and is glycosylated at multiple asparagine residues in consensus N-linked sequons. Unlike the heavily branched glycans found in eukaryotic N-linked glycoproteins, the modifying glycan structures in HMW1 are mono-hexoses or di-hexoses. Recent work demonstrated that the *H. influenzae* HMW1C protein is the glycosyltransferase responsible for transferring glucose and galactose to the acceptor sites of HMW1. An *Actinobacillus pleuropneumoniae* protein designated ApHMW1C shares high-level homology with HMW1C and has been assigned to the GT41 family, which otherwise contains only O-glycosyltransferases. In this study, we demonstrated that ApHMW1C has N-glycosyltransferase activity and is able to transfer glucose and galactose to known asparagine sites in HMW1. In addition, we found that ApHMW1C is able to complement a deficiency of HMW1C and mediate HMW1 glycosylation and adhesive activity in whole bacteria. Initial structure-function studies suggested that ApHMW1C consists of two domains, including a 15-kDa N-terminal domain and a 55-kDa C-terminal domain harboring glycosyltransferase activity. These findings suggest a new subfamily of HMW1C-like glycosyltransferases distinct from other GT41 family O-glycosyltransferases.

## Introduction

The *Haemophilus influenzae* HMW1 protein is a high-molecular weight non-pilus adhesin that mediates attachment to human epithelial cells, an essential step in the pathogenesis of *H. influenzae* disease [1]–[3]. HMW1 belongs to a family of proteins secreted via the two-partner secretion (TPS) pathway and requires a cognate outer membrane translocator protein called HMW1B for surface localization. HMW1 and HMW1B are prototype TPS proteins and are examples of TpsA and TpsB proteins, respectively. The HMW1 system requires an additional accessory protein called HMW1C for the fully functional system, a feature that is characteristic of a subset of TPS systems [3], [4]. In contrast to HMW1 and HMW1B, HMW1C lacks a signal sequence and remains in the cytoplasm.

In previous work, we established that HMW1 is a glycoprotein and undergoes glycosylation in the cytoplasm in a process that requires HMW1C [5]. Insertional inactivation of the *hmw1C* gene results in a change in apparent molecular mass of HMW1 (a 7–8 kDa decrease), partial degradation of HMW1, and a defect in tethering of HMW1 to the bacterial surface [5]. Examination of HMW1 proteolytic fragments by mass spectrometry identified 31 novel carbohydrate modification sites carrying 47 hexose units, corresponding to a molecular mass of ∼7.6 kDa [6]. All of the modified sites are asparagine residues, in all except one case in the conventional consensus sequence of N-linked glycans, namely Asn-X-Ser/Thr. Interestingly, the modifying carbohydrates at these sites are simple mono-hexose or di-hexose sugars rather than N-acetylated sugars, revealing an unusual carbohydrate modification and suggesting the presence of a glycosyltransferase with a novel enzymatic activity capable of transferring hexose moieties to asparagine residues [6]. Recently we established that HMW1C is the glycosyltransferase responsible for modifying HMW1 and is capable of transferring glucose and galactose from UDP-glucose and UDP-galactose to acceptor sites [7].

Carbohydrate modification of proteins is found in all domains of life and provides a mechanism for control of diverse cellular processes, including signal transduction, protein folding, sorting and stability [8], virus-cell interactions [9], and host immune responses [10]. In eukaryotes, N-linked protein glycosylation is the most common modification of secretory proteins and is coupled to protein translocation and folding. Since the realization that prokaryotes are able to glycosylate proteins, over 70 bacterial glycoproteins have been reported. The majority of these proteins are surface exposed and play a vital role in bacterial adhesion to host cells or evasion of host immunity. Studies of the glycan structures modifying bacterial glycoproteins have revealed that bacteria contain unusual and diverse carbohydrate units such as Pse and its derivatives [11]–[14]. The presence of simple mono-hexose and di-hexose structures at asparagine sites on HMW1 expands the recognized range of glycan structures on glycoproteins. Aside from the sugar structures of HMW1, the N-linked glycans on bacterial and non-bacterial glycoproteins are N-acetylated chitobiosyl core oligosaccharides attached to a well-established sequon of Asn-X-Ser/Thr. In contrast, O-linked glycans are either N-acetylated amino sugars or hexoses attached to Ser/Thr side chains, with no clear consensus sequence at the sites of attachment.

**Figure 1:**
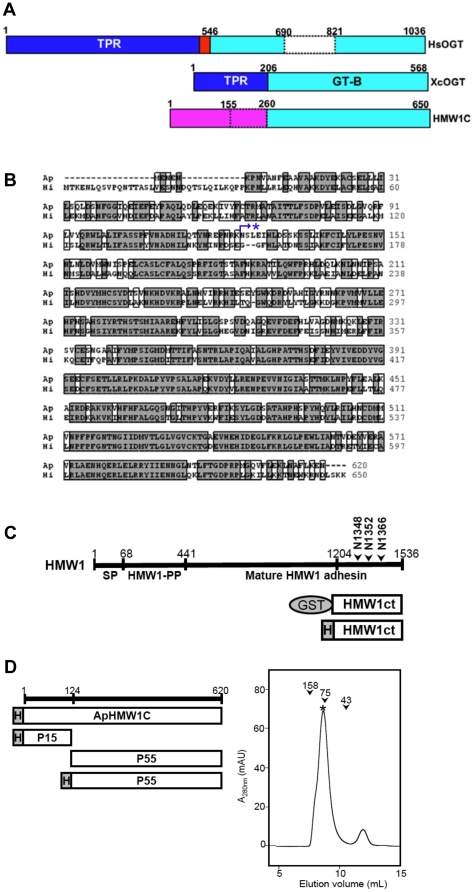
Representative GT41 members, HMW1C sequences, and schematics of recombinant proteins used in this study. (**A**) Domain organization of three GT41 members, including the human OGT (HsOGT), the *Xanthomonas campestris* OGT (XcOGT), and *Haemophilus influenzae* HMW1C protein (HMW1C). The TPR and GT domains are indicated in blue and cyan, respectively. Based on the XcOGT structure [17], [18], domain boundaries of HsOGT were assigned (the nucleus localization signal in red). Fly and mammalian OGTs have a large insertion (in white) within the GT domain. In HMW1C, the N-terminal domain (in magenta) is different from the TPR domains in HsOGT and XcOGT. HMW1C residues 155 and 260 correspond to ApHMW1C residue 125 (limited proteolysis boundary) and XcOGT residue 203 (boundary for GT), respectively. (**B**) The sequence alignment of HMW1C (Hi) with its ortholog from *A. pleuropneumoniae* (Ap). The protease cleavage site is indicated with star. (**C**) Schematics of HMW1 and acceptor protein constructs. The known domain organization of HMW1 is shown: SP, the signal peptide (residues 1–68); HMW1-PP, the HMW1 pro-piece (residues 69–441) containing the secretion domain; and the mature adhesin (residues 442–1536). Several constructs representing different regions of HMW1 were generated as GST-fusion proteins to serve as acceptor proteins. Based on assessment of solubility and stability of each protein in solution, the best substrate was HMW1ct. For the substrate HMW1ct, a His-tagged version was also produced. The N-glycosylation sites, N1348, N1352, and N1366, within HMW1ct are indicated. (**D**) Schematics of enzyme constructs. ApHMW1C (GenBank: ABN74719.1) and its two sub-domains (P15 and P55) identified from the analysis of limited proteolysis were produced as His-tagged proteins. An analytical gel filtration profile of purified ApHMW1C (marked with star) revealed a calculated molecular weight of ∼70 kDa, consistent with a monomer. The peak positions of molecular standards are indicated as arrowheads (aldolase, 158 kDa; conalbumin, 75 kDa; and ovalbumin, 43 kDa).

Analysis of sequenced genomes reveals a large number of predicted glycosyltransferases, amounting to ∼1–3% of ORFs in each genome [15]. The majority of these enzymes have low sequence similarity. Nonetheless, the CAZy database has provided very useful information on distinct groups of glycosyltransferases, classifying them into over 90 families [16]. Currently, HMW1C is classified into the GT41 family, which otherwise contains only O-linked GlcNAc transferases (OGT) [17], [18]. The OGT enzymes contain an N-terminal domain with so-called tetratricopeptide repeats (TPR) responsible for mediating recognition of a broad range of target proteins and a C-terminal glycosyltransferase domain responsible for binding and transferring UDP-GlcNAc to target proteins (Fig. 1A). With this information in mind, the observation that HMW1C lacks an N-terminal TPR domain and mediates N-linked glycosylation of HMW1 with simple hexoses raised questions about the specific structure and function of HMW1C-like proteins.

To understand the structural basis for HMW1 glycosylation, we have pursued structure and function studies of HMW1C. Since HMW1C has been recalcitrant to crystallization, we elected to explore an *Actinobacillus pleuropneumoniae* HMW1C homolog called ApHMW1C, which is ∼65% identical to HMW1C (Fig. 1B). *A. pleuropneumoniae* is a gram-negative, non-motile organism that belongs to the *Pasteurellaceae* family (like *H. influenzae*) and is the etiological agent of porcine pleuropneumonia, a severe contagious pulmonary disease of pigs that causes important economic losses in industrialized pig production worldwide [19]. In this study, we found that purified ApHMW1C has N-glycosyltransferase activity and transfers glucose and galactose to known asparagine glycosylation sites in HMW1, analogous to HMW1C. In addition, we demonstrated that ApHMW1C is able to complement a deficiency of HMW1C and mediates HMW1 glycosylation and adhesive activity in whole bacteria. Our findings suggest a new subfamily of N-glycosyltransferases distinct from other GT41 family O-linked GlcNAc transferases.

## Results

### Identification and purification of ApHMW1C

**Figure 2:**
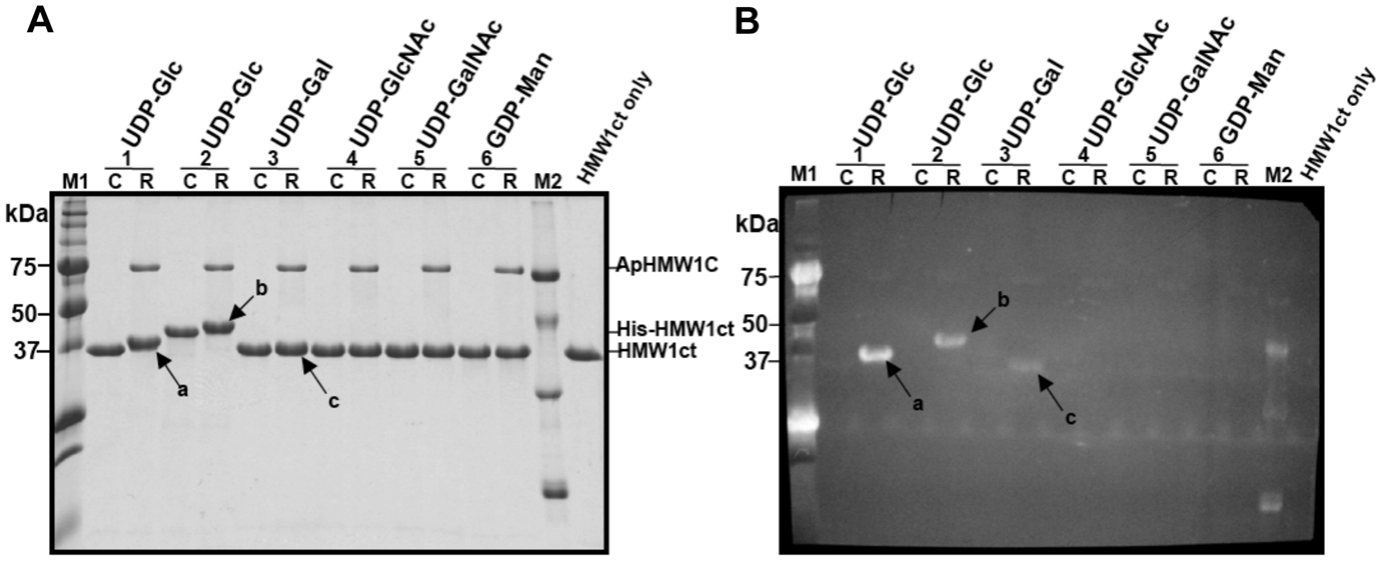
Glycosylation of HMW1ct by ApHMW1C. To define the donor substrate specificity of ApHMW1C, glycosylation reactions were carried out in the reaction buffer with (R-lanes) or without (C-lanes) ApHMW1C using different UDP (or GDP) activated sugars. HMW1ct (without fusion tag) was used as the acceptor protein (lanes 1, and 3 to 6). As a control, His-tagged HMW1ct (His-HMW1ct) was also tested in a reaction with UDP-glucose as the donor sugar (lanes 2). (**A**) After the glycosylation reactions, samples were separated by SDS-PAGE, and the gel was stained with Coomassie Blue. (**B**) In parallel, a duplicate gel was transferred to a PVDF membrane and subjected to a detection reaction using the GlycoProfile III Fluorescent Glycoprotein Detection kit (Sigma). Glycosylated HMW1ct proteins are indicated by arrows: ‘a’ and ‘c’ are glycosylated HMW1ct reacted with UDP-glucose and UDP-galactose, respectively, and ‘b’ is glycosylated His-HMW1ct reacted with UDP-glucose. The lanes labeled “M1,” “M2,” and “HMW1ct only” indicate pre-staining protein markers (Precision Plus Protein Standards, Bio-Rad), glycosylated protein markers (ProteoProfile PTM Marker, Sigma), and HMW1ct only as a control, respectively.

BLAST analysis of the *H. influenzae* HMW1C protein revealed a sequence from *Actinobacillus pleuropneumoniae* as the closest homolog, with 65% identity and 85% similarity overall (Fig. 1B). This *A. pleuropneumoniae* sequence is encoded by orf APL_1635 and is hereafter referred to as ApHMW1C. The *A. pleuropneumoniae* genome does not contain a locus encoding a TPS system (like the *hmw1* locus in *H. influenzae* that encodes HMW1, HMW1B, and HMW1C) or an isolated gene that encodes a predicted TpsA protein. Instead, the gene encoding ApHMW1C is adjacent to a gene cluster associated with sugar metabolism, namely APL_1631 (a homolog of glucosamine-fructose-6-phosphate aminotransferase), APL_1632 (a sugar metabolism transcriptional regulator), APL_1634 (putative glycosyltransferase), and APL_1635 (ApHMW1C). As a first step to examine the function of ApHMW1C, we overexpressed a His-tagged derivative using the pET45b vector backbone and *E. coli* BL21(DE3) (Fig. 1D). Subsequently we used a three-step purification protocol that included Ni^2+^-NTA affinity, anion-exchange, and gel filtration chromatography, yielding highly pure (>95%) recombinant protein. Based on the gel filtration profile of ApHMW1C (Fig. 1D), the calculated molecular weight was ∼70 kDa, consistent with the calculated molecular mass of 70,364 Da and the SDS-PAGE profile (Fig. 2A), indicating that ApHMW1C forms a monomer in solution.

### Generation of HMW1ct acceptor protein

The high level of homology between ApHMW1C and HMW1C raised the possibility that ApHMW1C has glycosyltransferase activity and glycosylates an *A. pleuropneumoniae* acceptor protein. Given that *A. pleuropneumoniae* lacks an obvious TpsA protein, we examined the ability of ApHMW1C to glycosylate the *H. influenzae* HMW1 adhesin. The mature HMW1 adhesin is a large protein ∼125 kDa in size (Fig. 1C), and the solubility of the recombinant protein is limited (data not shown). Therefore, we overexpressed fragments of HMW1 as GST fusion proteins and then examined the solubility of these fragments after removal of the GST moiety. Three fragments were found to be soluble when the GST moiety was present, but two of these fragments formed protein precipitates after the GST moiety was removed. The fragment corresponding to amino acids 1205–1536 at the C-terminal end of HMW1 (referred to as HMW1ct; Fig. 1C) was soluble with and without the GST moiety. The same fragment of HMW1 with a His tag at the N terminus (His-HMW1ct) was also soluble. Purification of HMW1ct (33.7 kDa) and His-HMW1ct (36.8 kDa) resulted in highly pure (>90%) protein as assessed by SDS-PAGE (Fig. 2A), suitable for functional studies using these preparations as the acceptor protein.

### ApHMW1C glycosylation of HMW1ct

To assess whether ApHMW1C is capable of glycosylating HMW1, we performed *in vitro* glycosylation assays using purified ApHMW1C, HMW1ct, and either UDP-glucose, UDP-galactose, or GDP-mannose, analogous to previous experiments with *H. influenzae* HMW1C [7]. In addition, we performed an *in vitro* assay using purified ApHMW1C, His-HMW1ct, and UDP-glucose. As shown in Fig. 2, in assays using UDP-glucose and UDP-galactose, we observed retarded gel mobility of HMW1ct and His-HMW1ct, consistent with glycan modification. In assays using UDP-GlcNAc or UDP-GalNAc, we observed no evidence of glycosylation (Figs. 2A and 2B), indicating that N-acetylated sugars are not suitable substrate carbohydrates for ApHMW1C. In addition, we observed no evidence of glycosylation using GDP-mannose. In control experiments, heat inactivation of ApHMW1C eliminated glycosylation activity (data not shown). These results are consistent with the glycan structure of HMW1 [6], indicating that ApHMW1C has substrate specificity similar to the *in vitro* and in *vivo* activity of HMW1C.

**Figure 3:**
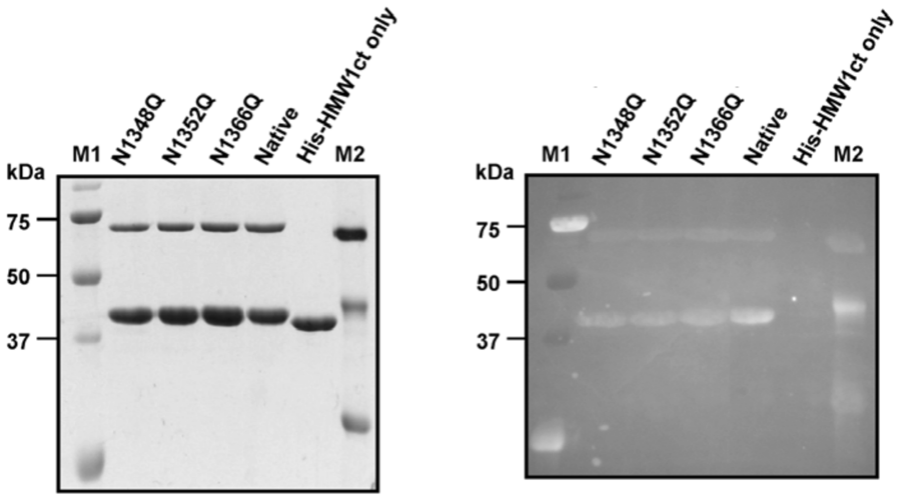
N-linked glycosylation of HMW1ct. The glycosylation reactions were carried out in standard conditions using single (N1348Q, N1352Q, N1366Q) mutants of His-HMW1ct as acceptor proteins and UDP-glucose as donor substrate. (**A**) After the glycosylation reaction, the samples were separated by SDS-PAGE, and the gel was stained with Coomassie Blue. (**B**) A duplicate gel was transferred to a PVDF membrane and subjected to a detection reaction using the GlycoProfile III Fluorescent Glycoprotein Detection kit (Sigma). The lanes labeled “Native” and “His-HMW1ct only” are control reaction samples with and without ApHMW1C, respectively. M1 is a pre-staining protein marker (Precision Plus Protein Standards, Bio-Rad), and M2 is a glycosylated protein marker (ProteoProfile PTM Marker, Sigma).

To determine whether ApHMW1C mimics HMW1C and modifies the same residues in HMW1ct, we used site-directed mutagenesis to convert Asn to Gln at the three known glycosylation sites in HMW1ct (N1348, N1352, and N1366) (Fig. 1C). The mutant proteins with a single mutation (N1348Q, N1352Q, or N1366Q) were over-expressed and purified and then analyzed for glycosylation intensity. As shown in Figure 3B, in *in vitro* glycosylation assays using the HMW1ct mutant proteins, ApHMW1C, and UDP-glucose, the band intensity of all HMW1ct mutant proteins was decreased compared to the intensity of wild type HMW1ct. These results establish that ApHMW1C mediates N-glycosylation of HMW1ct and functions analogously to HMW1C [7].

**Figure 4:**
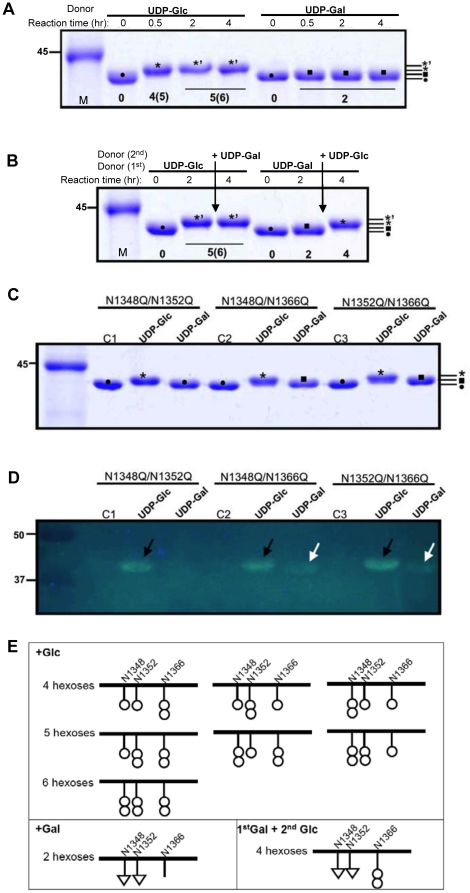
Specificity of HMW1ct glycosylation. The glycosylation reactions were carried out in standard conditions using His-HMW1ct as acceptor protein and UDP-glucose or UDP-galactose as donor substrate. (**A**) At each time point, an aliquot of the reaction was stopped by adding an equal volume of 2X SDS-PAGE sample buffer and followed by heating at 95°C for 4 min. (**B**) At each time point, SDS-PAGE samples were prepared as in A. However, two hrs after reaction with the first donor substrate, the second donor substrate was added to the reaction, as indicated. All samples were separated by 12% SDS-PAGE, and the gel was stained with Coomassie blue. The distinct shifts due to incorporated sugars are indicated by symbols (•, 0 hexose; ▪, 2 hexoses; ⋆, 4 or 5 hexoses; and ⋆’, 5 or 6 hexoses). (**C**) The glycosylation reactions were carried out in standard conditions using double mutants of His-HMW1ct (N1348Q/N1352Q, N1348Q/N1366Q, and N1352Q/N1366Q) by ApHMW1C using UDP-glucose or UDP-galactose as donor substrates. C1, C2, and C3 indicate control reactions without ApHMW1C. Samples were separated by 12% SDS-PAGE and were stained with Coomassie blue. (**D**) In parallel, a duplicated gel was transferred to a PVDF membrane and subjected to a detection reaction using the GlycoProfile III Fluorescent Glycoprotein Detection kit (Sigma). The glycosylated proteins by UDP-glucose or by UDP-galactose are indicated by arrows. (**E**) Model of hexose modifications at Asn-1348, Asn-1352, and Asn-1366.

To investigate the apparent difference in gel shift and fluorescence intensity between the reaction with UDP-glucose and the reaction with UDP-galactose (Fig. 2), we monitored the progression of His-HMW1ct glycosylation by ApHMW1C. With both UDP-glucose and UDP-galactose, the modification reached a maximal gel shift within 2 hrs (Fig. 4A). Subsequently, we examined the effect of the order of addition of hexose sugars on glycosylation of HMW1ct. As shown in Fig. 4B, when adding UDP-galactose to the UDP-glucose saturated reaction, no change was observed, indicating that galactose is not further incorporated into the modified HMW1ct. On the other hand, adding UDP-glucose to the UDP-galactose saturated reaction resulted in a higher intermediate species of the modified protein, but did not restore to the maximal modification profile observed with UDP-glucose alone, suggesting that galactose may not be incorporated at all three Asn sites. To test this possibility, three HMW1ct variants with a double mutation (N1348Q/N1352Q, N1348Q/N1366Q, N1352Q/N1366Q) were over-expressed and purified and then analyzed for glycosylation intensity after incubation with ApHMW1C and either UPD-glucose or UDP-galactose. As shown in Fig. 4C, 4D, and 4E, glucose was incorporated at all three sites, whereas galactose was incorporated at Asn-1348 and Asn-1352 but not at Asn-1366. These results are consistent with a recent analysis of HMW1C glycosylation of HMW1 [7] and demonstrate that modification with galactose occurs at a restricted number of Asn sites.

### ApHMW1C glycosylation of HMW1

**Figure 5:**
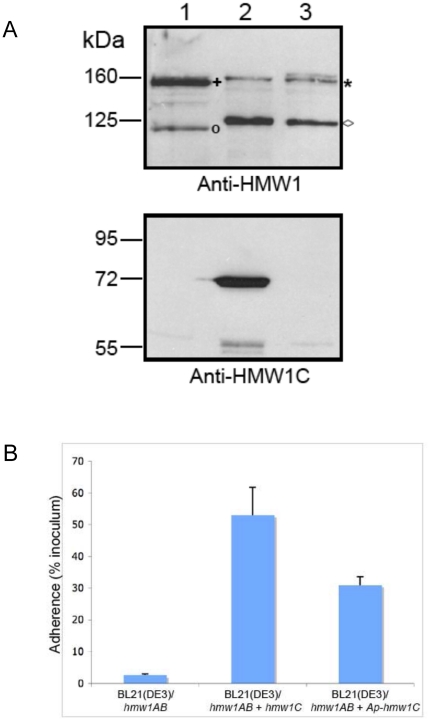
Ability of ApHMW1C to complement a deficiency in HMW1C. Panel **A** shows Western immunoblots of whole cell sonicates of *E. coli* BL21(DE3)/pACYC-HMW1ΔC (lane 1), *E. coli* BL21(DE3)/pACYC-HMW1ΔC + pET45b-HMW1C (lane 2), and *E. coli* BL21(DE3)/pACYC-HMW1ΔC + pET45b-ApHMW1C (lane 3). Lane 1 contains twice as much protein as loaded in lanes 2 and 3 to increase the visibility of the non-glycosylated HMW1 species. The blot in the upper panel was performed with a guinea pig antiserum reactive with HMW1, and the blot in the lower panel was performed with a guinea pig antiserum reactive with *H. influenzae* HMW1C. The asterisk indicates the glycosylated HMW1 pro-protein, and the plus sign indicates the non-glycosylated HMW1 pro-protein. The diamond indicates the glycosylated HMW1 mature protein, and the circle indicates the non-glycosylated HMW1 mature protein. Panel **B** shows *in vitro* adherence results comparing adherence by *E. coli* BL21(DE3)/pACYC-HMW1ΔC (*hmw1AB*), *E. coli* BL21(DE3)/pACYC-HMW1ΔC + pET45b-HMW1C (*hmw1AB* + *hmw1C*), and *E. coli* BL21(DE3)/pACYC-HMW1ΔC + pET45b-ApHMW1C (*hmw1AB* + *Aphmw1C*) to Chang epithelial cells. Bars and error bars represent mean and standard error measurements from a representative assay with measurements performed in triplicate.

To extend our *in vitro* results and examine whether ApHMW1C is capable of glycosylating full-length HMW1, we introduced ApHMW1C into *E. coli* BL21(DE3) harboring the *hmw1* locus with a deletion of *hmw1C* (*hmw1AB*). As controls, we examined *E. coli* BL21(DE3)/*hmw1AB* and *E. coli* BL21(DE3)/*hmw1AB* + *hmw1C*. As shown in Figure 5A, ApHMW1C was capable of complementing a deletion of *hmw1C*, restoring normal glycosylation, stability, and processing of HMW1. In addition, ApHMW1C was able to restore HMW1 adhesive activity, as measured by bacterial adherence to cultured epithelial cells (Fig. 5B).

### Catalytic properties of ApHMW1C

**Figure 6:**
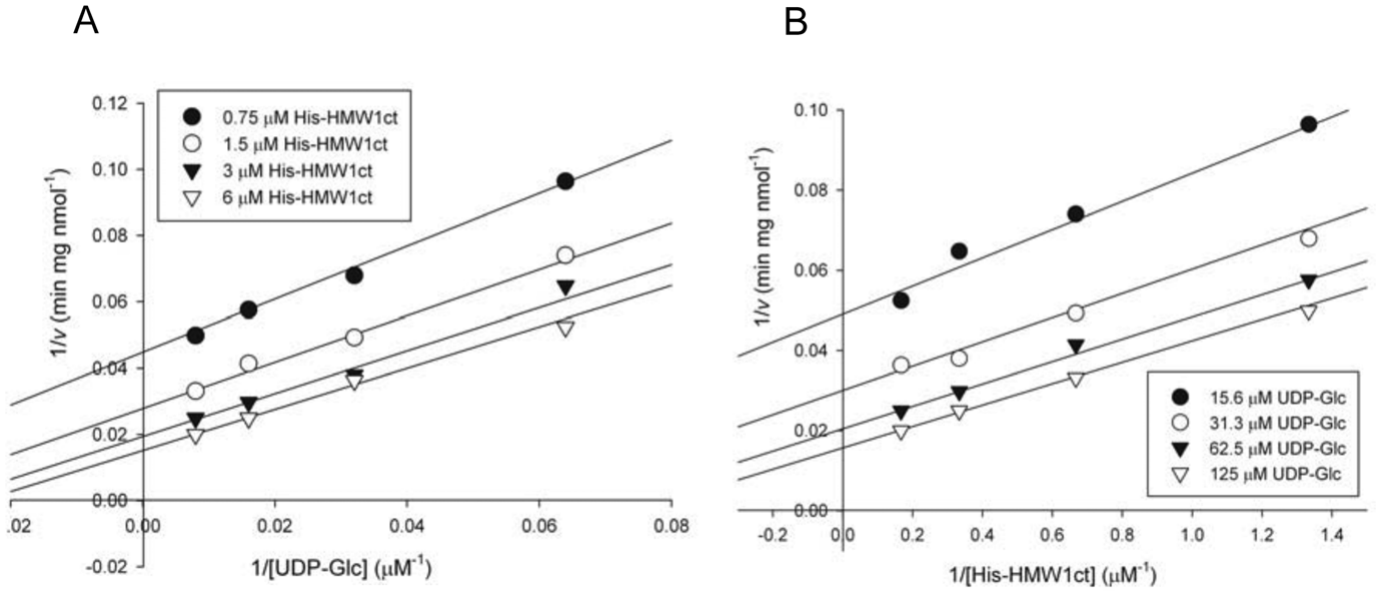
Initial velocity of ApHMW1C. (**A**) Double reciprocal plots of the initial velocity of ApHMW1C as a function of UDP-glucose concentration at the fixed His-HMW1ct concentrations, as indicated (inset). (**B**) Double reciprocal plots of the initial velocity of ApHMW1C as a function of His-HMW1ct concentration at the fixed UDP-glucose concentrations, as indicated (inset). The true K_m_ values of UDP-glucose and His-HMW1ct corresponded to 54.5 µM and 2.3 µM, respectively, as obtained by eq. 2.

To delineate the catalytic properties of ApHMW1C, the kinetics of the glycosyltransferase reaction were monitored using a continuous coupled spectrophotometric assay. In this assay, formation of UDP was measured by oxidation of NADH using pyruvate kinase and lactate dehydrogenase as coupling enzymes [20]. His-HMW1ct was used as the acceptor protein, and UDP-glucose and UDP-galactose were used as donor substrates. ApHMW1C activity was not influenced by the addition of Mg^2+^ and Mn^2+^ or by the presence of excess EDTA according to gel shift assays (data not shown), suggesting that activity is independent of exogenous metal ions. Despite these results, we included K^+^ and Mg^2+^ in standard assay conditions because of their importance as metal cofactors for the coupling enzymes. The apparent K_m_ and V_max_ values for reactions with UDP-glucose and with UDP-galactose were determined by fitting initial rate data to the Michaelis-Menten equation (eq. 1), as summarized in Table 1. This analysis demonstrated that UDP-glucose is preferred over UDP-galactose as a donor substrate (k_cat_/K_m_  = 3.3×10^3^ M^−1^s^−1^ vs. k_cat_/K_m_  = 5.2×10^2^ M^−1^s^−1^), consistent with our results using the glycan detection method. For the glucose transfer reaction, the true K_m_ values of two substrates (sugar and protein) were determined by measuring initial rates at concentrations ranging from 15.6 to 125 µM for UDP-glucose and from 0.75 to 6 µM for HMW1ct. The pattern of double-reciprocal Lineweaver-Burk plots for initial rates suggests a sequential mechanism, since all lines were linear and converged at the left side of the y-axis (Fig. 6). A ping-pong mechanism can be ruled out, since it would be expected to generate parallel lines in Lineweaver-Burk plots. Moreover, ApHMW1C could not auto-hydrolyze the UDP moiety from UDP-glucose in the absence of an acceptor protein (data not shown), suggesting that ApHMW1C may adopt the ordered or random sequential mechanism.

**Table 1:** Kinetic parameters of ApHMWC and its derivative proteins.

Parameter a	*K_m_*	*V_max_*	*k_cat_*	*k_cat_/K_m_*
	(µM)	(nmol min^−1^ mg^−1^)	(s^−1^)	(M^−1^s^−1^)
*UDP-Glc*				
ApHMW1C	38.9±5.8	108.8±4.0	0.130±0.005	(3.3±0.8) ×10^3^
P55	67.3±3.8	141.0±2.4	0.132±0.002	(2.0±0.6) ×10^3^
P15	ND	ND	ND	ND
*UDP-Gal*				
ApHMW1C	147.5±24.8	64.3±4.3	0.077±0.005	(5.2±2.1) ×10^2^
P55	206.8±50.8	44.2±4.9	0.041±0.005	(2.0±0.9) ×10^2^
P15	ND	ND	ND	ND

a These are apparent values, determined by varying the concentration of one substrate (sugar donor substrate) at a fixed concentration of the second (protein acceptor).

### Probing the domain structure of ApHMW1C

**Figure 7:**
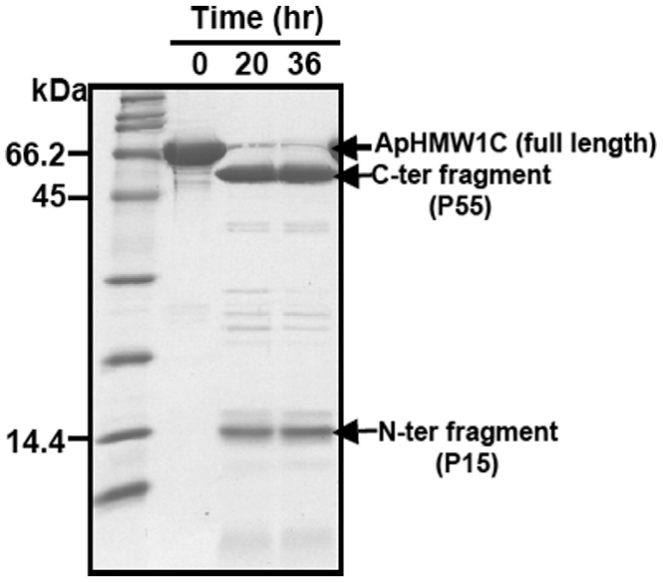
Probing functional domains of ApHMW1C. SDS-PAGE of limited proteolysis identified two stable fragments from ApHMW1C. Proteolysis reactions were carried out as described in experimental procedures. Shown is the reaction with trypsin:ApHMW1C (w/w) ratio of 1∶100 at 6°C.

To experimentally determine the domain structure of ApHMW1C and gain general insights into the architecture of HMW1C-like proteins, we performed limited proteolysis of ApHMW1C (Fig. 7). Using 1∶100 and 1∶50 molar ratios of trypsin to ApHMW1C, reaction mixtures were monitored and revealed a gradual disappearance of full-length ApHMW1C and appearance of two major fragments. N-terminal sequencing established that the small fragment (referred to as P15; Fig. 1D) corresponds to the N-terminal domain of ApHMW1C and that the large fragment (referred to as P55; Fig. 1D) corresponds to the C-terminal domain of ApHMW1C starting with Asn-125. To investigate the functional roles of these domains, we produced each domain as a recombinant His-tagged protein. The P15 domain could be purified as a soluble protein, but the P55 domain was completely insoluble. Interestingly, P15 and P55 could be co-purified when we performed scale-up preparative limited proteolysis followed by chromatography. Based on these observations, the two domains appear to be associated and the N-terminal P15 domain appears to be important for the stability and/or solubility of the recombinant ApHMW1C. Next, we investigated the glycosylation activity of P15 and P55 (Fig. 1D). The P15 fragment was purified by affinity and gel filtration chromatography and had no detectable activity with either donor substrate. Given that His-tagged P55 was insoluble, the P55 fragment was obtained by preparative limited trypsin proteolysis followed by purification using ion exchange and gel filtration chromatography. Purified P55 showed 60% catalytic efficiency (k_cat_/K_m_  =  2.0×10^3^ M^−1^s^−1^) relative to native ApHMW1C (k_cat_/K_m_  =  3.3×10^3^ M^−1^s^−1^) using UDP-glucose and 38% catalytic efficiency (k_cat_/K_m_  = 2.0×10^2^ M^−1^s^−1^) relative to native ApHMW1C (k_cat_/K_m_  = 5.2×10^2^ M^−1^s^−1^) using UDP-galactose (Table 1). These results demonstrate that P55 (*i.e.* the fragment generated by deleting the P15 region from ApHMWC) has reduced enzyme activity.

## Discussion

In this study, we characterized the enzyme properties of an *A. pleuropneumoniae* HMW1C homolog designated ApHMW1C. Both HMW1C and ApHMW1C have been assigned to the GT41 family of glycosyltransferases in the CAZy database. The GT41 family includes both bacterial and eukaryotic proteins and is characterized by large O-linked GlcNAc transferases with a long N-terminal extension containing so-called tetratricopeptide repeats (the TPR domain). To date the only member of the GT41 family that has been functionally characterized is the conserved mammalian O-linked GlcNAc transferase referred to as OGT. This enzyme modifies the side chains of serine and threonine residues of nuclear and cytosolic proteins with GlcNAc [21]–[24]. The N-terminal TPR domain of OGT mediates the recognition of a broad range of target acceptor proteins, and the C-terminal region contains the glycosyltransferase catalytic domain [17], [18], [25]. OGT-mediated glycosylation is a dynamic process in which GlcNAc is rapidly added and removed compared with the lifetime of the acceptor protein, analogous to protein phosphorylation. Given that HMW1C and ApHMW1C catalyze N-glycosylation and transfer simple hexose sugars to multiple asparagine sites of the HMW1 adhesin [6], HMW1C-like proteins clearly differ from previously reported enzymes in the GT41 family.

Historically, glycosyltransferases have been very difficult to study biochemically. These enzymes are notoriously unstable, often containing flexible loops and domains and commonly associating with other interacting proteins [26]. Thus far, we have been unable to obtain sufficiently high concentrations of HMW1C in solution for crystallography studies. As an alternative approach, we turned to ApHMW1C and found that we were able to purify high quantities that remained soluble. At the amino acid sequence level, ApHMW1C and HMW1C are ∼65% identical and ∼85% similar. However, the *A. pleuropneumoniae* genome does not have a locus corresponding to a TPS system (*e.g.*, the *hmw1* locus in *H. influenzae* encoding HMW1, HMW1B, and HMW1C) or an isolated gene that encodes a predicted TpsA protein. Instead, the gene encoding ApHMW1C is adjacent to a gene cluster with orfs involved in sugar metabolism. This observation suggests the possibility that HMW1C-like proteins have evolved in certain organisms to transfer sugars to protein acceptors that are not necessarily TpsA proteins in TPS systems.

In the absence of a known acceptor protein for ApHMW1C in *A. pleuropneumoniae*, we addressed the specific function of ApHMW1C using HMW1 as an acceptor protein. In *in vitro* assays containing ApHMW1C, a C-terminal fragment of HMW1 (HMW1ct), and either UDP-glucose or UDP-galactose, we observed evidence of glycosylation of HMW1ct via both the Glycoprofile III Fluorescent Glycoprotein Detection kit and a noticeable shift in mobility on SDS-PAGE gels. To extend these findings, we examined the specificity of ApHMW1C glycosylation activity by mutagenesis, focusing on the three Asn residues in HMW1ct that are known to be glycosylated by HMW1C [6]. Individual mutations of each Asn site (N1348Q, N1352Q, N1366Q) resulted in a clear reduction in fluorescence intensity (Fig. 3).

Based on the observed differences in the gel shift and the fluorescence intensity between the reaction with UDP-glucose and the reaction with UDP-galactose, two hexoses might be added at different sites in the acceptor protein and/or in different forms such as mono- or di-hexoses [6]. These possibilities were addressed by monitoring glycosylation as a function of time. Using our assay conditions, the 2hr reaction time appears to be sufficient for the maximal modification with both donor substrates. Interestingly, we observed only one species of the modified protein with UDP-galactose but two species with UDP-glucose in the course of the reaction (Fig. 4A), indicating that the transfer of glucose is more complex than the transfer of galactose. Indeed, the results of the reaction order of hexose sugars indicated that glucose can be incorporated at site(s) where galactose cannot and that glucose can be further incorporated to produce di-hexoses (Fig. 4B), as originally observed with HMW1C glycosylation of HMW1 [7]. Using HMW1ct derivatives with double mutations, we established that ApHMW1C can transfer the glucose moiety from UDP-glucose to all three of the documented glycosylation Asn sites within HMW1ct but can transfer the galactose moiety from UDP-galactose to only two of the three Asn sites. These observations demonstrate that UDP-glucose is the major donor substrate for ApHMW1C, resulting in transfer of glucose to either an Asn residue or to another glucose modifying an Asn residue, consistent with findings with HMW1C/HMW1.

HMW1C and ApHMW1C share 42–65% identity and 58–85% similarity with proteins in a number of other gram-negative bacterial pathogens, including the enterotoxigenic *E. coli* EtpC protein and predicted proteins in *H. ducreyi*, *Yersinia* spp., *Mannheimia* spp., and *Burkholderia* spp, among others [7]. Considering the results with ApHMW1C and HMW1C, HMW1C-like proteins catalyze N-glycosylation of an acceptor protein and appear to have acquired a secondary O-glycosylation activity that involves transferring a donor sugar to an acceptor sugar, forming di-hexoses on glycoproteins. The capacity to generate di-hexose O-glycosyidic bonds may explain why HMW1C-like proteins share similarity with GT41 O-glycosyltransferases within the C-terminal domain (Fig. 1A). However, the fact that OGTs transfer GlcNAc from UDP-GlcNAc to the side chain hydroxyl group of an amino acid (Ser or Thr) rather than transferring glucose from UDP-Glc to the hydroxyl group of glucose as in HMW1C-like proteins may explain why the sequence identities between HMW1C-like enzymes and OGT-like enzymes are marginal. One obvious common feature of these two groups of GT41 proteins is that both bind sugars donors that are activated by UDP, requiring a binding pocket that is formed by the C-terminal half of the GT-B domain (Fig. 1A) [17], [18].

Using a continuous coupled spectrophotometric assay to assess enzymatic activity, we observed that ApHMW1C is an efficient N-glycosyltransferase capable of transferring glucose and galactose moieties to multiple asparagine residues of HMW1 with k_cat_/K_m_ values of 3.3×10^3^ M^−1^s^−1^ and 5.2×10^2^ M^−1^s^−1^, respectively. Like the majority of enzymes in the GT-B superfamily of glycosyltransferases [27]–[32], ApHMW1C showed metal-independent behavior (data not shown), suggesting that ApHMW1C has a GT-B fold, consistent with assignment to the GT41 family. Based on the results of limited proteolysis, ApHMW1C appears to contain two domains, namely a minor N-terminal domain (P15) and a major C-terminal domain (P55). Regardless of donor substrates, the P55 domain had lower catalytic efficiency. At this point, it is unclear whether or not the small N-terminal domain contributes to enzyme efficiency. In order to understand the structural basis for the enzyme mechanisms and specificities of O-linked GlcNAc transferase and HMW1C-like proteins, obtaining the crystal structure of an HMW1C-like glycosyltransferase is highly desirable.

In bacteria, only limited information is available about the structures of protein-linked glycans, the detailed process of protein glycosylation, and the functional implications of glycosylation. However, given that nearly all of the known glycoproteins are surface-exposed, glycosylation appears to have an important role in pathogenesis. Recently characterized functions of bacterial protein glycosylation include adhesion, protection against proteases, antigenic variation, and evasion of immunity [5], [12], [33]–[38]. Among bacterial glycoproteins, *H. influenzae* HMW1 and diarrheagenic *E. coli* AIDA-I are particularly noteworthy, representing two branches of the type V secretion system. HMW1 is a TpsA protein secreted by the TPS pathway, and AIDA-I is an autotransporter protein. It is known that glycosylation of HMW1 protects against premature degradation and is required for HMW1 tethering to the bacterial surface, a prerequisite for HMW1-mediated adherence. Similarly, glycosylation of AIDA-I is required for normal conformation and for AIDA-I mediated adhesion to epithelial cells [39]. In considering the secretion pathway, glycosylation might play a role in secretion of exoproteins, reminiscent of the eukaryotic secretory pathway. Of note, HMW1 contains N-linked hexoses at over 30 sites along the mature protein, whereas AIDA-I contains O-linked heptoses at ∼16 sites along the protein [39]. The enzyme responsible for glycosylation of AIDA-I has not been studied. Seemingly, these bacterial glycoproteins secreted by the type V secretion pathway share common features such as simple glycan structures (hexoses in HMW1 vs. heptoses in AIDA-I) and multiple site modification. Yet, these proteins are distinct in that HMW1 has N-linked glycans and AIDA-I has O-linked glycan. In order to further our knowledge of the mechanisms and functional implications of glycosylation of proteins secreted via the type V secretion pathway, structural and functional studies on the corresponding enzyme systems are critical. Along these lines, our functional characterization of ApHMW1C is an important advance.

In summary, HMW1C and ApHMW1C add simple hexose sugars to asparagine sites of acceptor proteins and appear to represent a new subfamily of bifunctional glycosyltransferases (N-linkage to protein acceptor and O-linkage to glucose acceptor), distinct from GT41 family O-glycosyltransferases. Determination of the structures of HMW1C and ApHMW1C will provide a major advance in understanding how members of this family glycosylate HMW1 and other acceptor proteins.

## Materials and Methods

### Materials

Restriction enzymes were purchased from New England Biolabs. *Pfu* DNA polymerase and T4 DNA ligase were purchased from Stratagene and Promega, respectively. Primers used for PCR were synthesized by IDT. Unless indicated otherwise, chemicals were purchased from Sigma, including UDP- and GDP-sugars, β-NADH and phosphoenolpyruvate (PEP), and coupling enzymes (LDH/PK).

### Cloning and expression

The gene encoding ApHMW1C (GenBank: ABN74719.1) was amplified by PCR from genomic DNA of *Actinobacillus pleuropneumoniae* (purchased from ATCC: Cat no. 27088D-5) using primers 1 and 2 (Table 2) and was inserted into *Kpn*I/*Sac*I-digested pET45b (Novagen) to express an N-terminal His-tag protein. The gene encoding *H. influenzae* HMW1C was amplified by PCR from pHMW1-15 [40] using primers 3 and 4 (Table 2) and was inserted into *Kpn*I/*Sac*I-digested pET45b. The DNA fragment encoding the C-terminal end of *Haemophilus influenzae* HMW1 corresponding to amino acids 1205–1536 (HMW1ct, Fig. 1C) was amplified by PCR from pHMW1-15 [40] using primers 5 and 6 (Table 2) and was inserted into *Bam*HI/*Xho*I-digested pGEX-6p-1 (GE Healthcare) and *Bam*HI/*Xho*I-digested pET28a (Novagen). Mutants of HMW1ct were generated using the QuikChange II site-directed mutagenesis kit (Stratagene) and mutagenic primer sets (Table 2) according to the manufacturer's instructions.

**Table 2:** Primers used for cloning, site-directed mutagenesis, and sequence analysis.

Protein names	Primer #	Primer sequences (5′ → 3′)
ApHMW1C	1	cgg ggt acc atg gaa aac gaa aat aaa ccg aat
	2	cgc gag ctc tta att ttc ttt tag gaa cgc att t
HMW1C	3	gtg ggt acc atg aca aaa gaa aat tta caa agt g
	4	gtg gag ctc tta ttt ttt actcaagtgcttccg
HMW1ct	5	gcg gga ttc gca aat agc ggt gca tta acc
	6	ggc ctc gag cta ccg ccc gtt atc agc gat
HMW1ct variants		
HMW1ct_N1348Q_	7	gca gga agt att aat gcc gcc **cag** gtg aca cta aat act aca ggc
HMW1ct_N1352Q_	8	aat gcc gcc aat gtg aca cta **cag** act aca ggc act tta act acc
HMW1ct_N1348Q/N1352Q_	9	aat gcc gcc **cag** gtg aca cta **cag** act aca ggc act tta act acc
HMW1ct_N1366Q_	10	acc gtg aag ggt tca aac att **cag** gca acc agc ggt acc ttg gtt

### Protein purification

To purify His-tagged proteins, the pET-based plasmids containing the genes encoding ApHMW1C, ApHMW1C-HMW1C hybrids, and HMW1ct were expressed in *E. coli* BL21(DE3) (Stratagene). Bacteria were grown to an OD_600_ of 0.8, and expression was induced with 0.2 mM IPTG at room temperature for 2 hrs. Subsequently, bacterial cells were harvested, resuspended in buffer-A (20 mM Tris-HCl, pH 8.0, 250 mM NaCl, 5 mM β-mercaptoethanol) containing 10 mM imidazole, and disrupted using sonication. After centrifugation at 40,000x*g* for 20 min (Sorvall), supernatants were subjected to a binding reaction with Ni^2+^-NTA (Qiagen) resin for 30 min in a batch procedure. Protein-resin complexes were then packed onto a column and washed with buffer-A containing 20 mM imidazole. The proteins were eluted using a step gradient method including 40, 60, 100, 300 and 500 mM imidazole in buffer-A. Fractions containing target proteins were diluted five times with buffer-B (20 mM Tris-HCl pH 8.0), loaded onto a HiTrap-Q column (GE Healthcare), and eluted with a linear gradient of 50 mM–1M NaCl in buffer-B using an AKTA FPLC system (GE Healthcare). The peak fractions were combined, concentrated, and further purified using a Superdex 75 10/300 GL gel filtration column (GE Healthcare) equilibrated with buffer-C containing 50 mM HEPES, pH 7.0, 200 mM NaCl, 0.1 mM EDTA, and 5% glycerol.

To purify GST fusion proteins, constructs were expressed in *E. coli* BL21 (Stratagene). Bacteria were grown to an OD_600_ of 0.8, and expression was induced with 0.4 mM IPTG at room temperature for 2 hrs. Bacterial cells were harvested and resuspended in PBS containing 1 mM DTT. Following cell lysis using sonication, cell lysates were centrifuged at 40,000x*g* for 20 min, and the supernatant was added to GSH-Sepharose 4 fast flow beads (GE Healthcare). After binding for 2 hrs at 4°C and washing with PBS, GST-fusion proteins were eluted with 10 mM GSH in 50 mM Tris-HCl, pH 8.0. The GST moiety was cleaved via on-column digestion with 80 units PreScission protease (GE Healthcare) in 4 mL of cleavage buffer (50 mM Tris-HCl, pH 7.0, 150 mM NaCl, 1 mM EDTA, and 1 mM DTT) overnight at 4°C [41]. Eluted proteins were further purified using a HiTrap-Q column as described above. The protein concentration was determined by the Bradford assay following the manufacturer's instructions (Bio-Rad).

### Glycan detection

To detect glycosylation of the HMW1ct acceptor protein *in vitro*, ApHMW1C in a final concentration of 1.6 µM was added to a 100 µL reaction mixture containing 50 mM HEPES, pH 8.0, 50 mM KCl, 5 mM MgCl_2_, 0.5 mM UDP- or GDP-sugar, and 6.3 µM HMW1ct. The mixtures were incubated at room temperature for 2 hrs and were then added to an equal volume of 2X SDS-PAGE sample buffer and incubated at 95°C for 4 min. The samples were separated on a 15% SDS-PAGE gel, transferred to a PVDF membrane using a Semi-Dry Transfer Cell (Bio-Rad), and detected by a GlycoProfile III Fluorescent Glycoprotein Detection kit (Sigma) according to the manufacturer's instructions.

### Enzyme assays and data analysis

Glycosyltransferase activity was measured using a continuous coupled spectrophotometric assay for UDP formation in standard assay conditions [20]. Reaction mixtures containing 50 mM HEPES pH 8.0, 50 mM KCl, 5 mM MgCl_2_, 0.5 mM UDP-glucose, 3.2 µM His-HMW1ct, 0.2 mM β-NADH, 2 mM phosphoenolpyruvate (PEP), 2.5 U lactate dehydrogenase (LDH), and 1.75 U pyruvate kinase (PK) were pre-incubated at room temperature for 10 min. Reactions were started by adding ApHMW1C to a final concentration of 0.9 µM, and the initial glycosylation rates were measured by monitoring the decrease of absorbance at 340 nm for 1 min. One unit was defined as the amount of enzyme that produced 1 nmol of UDP per minute in standard assay conditions. Initial rates (v) from the kinetic studies were plotted as 1/v *vs.* 1/[substrate concentration]. The data were fit to the appropriate rate equations using the Enzyme Kinetic Module version 1.3 integrated into SigmaPlot ver 10.0.1 (Systat). The apparent K_M_ and V_max_ values were determined by fitting the initial rates to the Michaelis-Menten equation (eq. 1). Data conforming to a sequential mechanism were fit to the Random Bi-Bi equation (eq. 2).
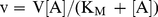
(1)

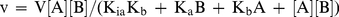
(2)


In eqs 1–2, A and B are the concentration of the substrates, v and V represent initial and maximum rates, respectively, K_a_ and K_b_ are Michaelis constants for A and B, respectively, and K_ia_ is the inhibition constant for substrate A.

### Adherence assays

Adherence assays were performed with Chang epithelial cells (human conjunctiva; ATCC CCL 20.2) (Wong-Kilbourne derivative clone 1-5c-4) as described previously [42]. Bacteria were prepared by inoculating LB broth containing antibiotics to select for the relevant plasmids and incubating overnight, then diluting 1∶10 in fresh LB broth with antibiotics and incubating for 90 min, then adding IPTG to a final concentration of 0.03 mM and incubating for 2 hrs to induce expression of ApHMWC or HMW1C. Percent adherence was calculated by dividing the number of adherent colony-forming units by the number of inoculated colony-forming units. All strains were examined in triplicate, and assays were repeated three times.

### Strains and protein analysis for *in vivo* functional assays

To test whether ApHMW1C is active *in vivo*, the following strains were used: BL21(DE3) expressing pACYC-HMW1ΔC (*hmw1AB*) [5], BL21(DE3) expressing pACYC-HMW1ΔC + pET45b-ApHMW1C, and BL21(DE3) expressing pACYC-HMW1ΔC + pET45b-HMW1C. Whole cell sonicates were prepared by suspending bacterial pellets in 10mM HEPES, pH 7.4 and sonicating to clarity. Proteins were resolved by SDS-PAGE using 7.5% polyacrylamide gels. Western blots were performed using antiserum GP96, a guinea pig polyclonal antiserum against the HMW1 and HMW2 proteins [43].

### Limited proteolysis of ApHMW1C

Protease digestions were performed as described previously [44] with the following modifications. For each 100 µl reaction, 100 µg of a purified ApHMW1C sample was incubated with trypsin (Roche Dignostics) in 20 mM Tris-HCl (pH 7.5), NaCl 100 mM, and 1 mM EDTA (pH 8). Reactions were performed at trypsin:ApHMW1C (w/w) ratios of 1∶50, 1∶100, and 1∶500 by incubating in a heat block at 37°C for up to 3 hrs or at 6°C for up to 36 hrs. At each time point, aliquots of the reactions were stopped by the addition of 2X SDS-PAGE loading buffer followed by boiling for 4 min. The samples were analyzed by SDS-PAGE, transferred to a PVDF membrane, and submitted for N-terminal sequencing (Midwest Analytical Inc., St. Louis).
